# Symptom distress and suicidal ideation among Chinese ovarian cancer patients: A moderated mediation model of depression and suicide resilience

**DOI:** 10.3389/fpsyg.2023.1073995

**Published:** 2023-02-21

**Authors:** Jie Chen, Yinying Zhang, Fang Cheng, Jinzhi Xie, Keke Zhang, Deying Hu

**Affiliations:** ^1^Department of Nursing, Union Hospital, Tongji Medical College, Huazhong University of Science and Technology, Wuhan, China; ^2^School of Nursing, Tongji Medical College, Huazhong University of Science and Technology, Wuhan, China; ^3^Cancer Center, Union Hospital, Tongji Medical College, Huazhong University of Science and Technology, Wuhan, China

**Keywords:** symptom distress, suicidal ideation, depression, suicide resilience, ovarian cancer

## Abstract

**Objective:**

The aim of this study was to examine whether depression mediates the relationship between symptom distress and suicidal ideation in Chinese patients with ovarian cancer, and whether this mediating effect was moderated by suicide resilience.

**Methods:**

From March to October 2022, this cross-sectional study was performed in a three Grade 3A hospital and an oncology specialty hospital in Wuhan, Hubei Province, China. Ultimately, 213 ovarian cancer patients completed anonymous self-report. Bootstrapping method was used for regression analysis to test the mediating and moderating effects.

**Results:**

Among the 213 participants, 29.58% (*n* = 63) exhibited significant suicidal ideation. Symptom distress was positively associated with suicidal ideation, and depression partially mediated this relationship. Suicide resilience moderated the relationship between depression and suicidal ideation. In ovarian cancer patients with low suicide resilience, the effect of symptom distress on suicidal ideation through depression was greater, while in patients with high suicide resilience, this effect was attenuated.

**Conclusion:**

Our study suggests that symptom distress could be more likely to lead to suicidal ideation as depression levels increase in ovarian cancer patients. Fortunately, suicide resilience could attenuate this negative effect.

## 1. Introduction

Cancer is one of the leading causes of human death in the world. The shock of diagnosis and subsequent treatment may cause psychological and physical distress, affecting the quality of life and even the will to live (Antoni et al., 2022; Ma et al., 2022). A recent meta-analysis involving more than 22 million cancer patients showed a nearly twofold (1.85-fold) increase in suicide death rate compared to the general population (Heinrich et al., 2022). Suicidal ideation is a cognitive construct associated with suicide risk (Kolva et al., 2020) and a marker of excruciating psychological distress, which includes thinking about, considering, or preparing suicide (Klonsky et al., 2016). In a more recent review, the incidence of suicidal ideation in cancer patients ranged from 0.7 to 46.3% (Kolva et al., 2020). Previous suicidal ideation is a major risk factor for future suicidal behavior (Large et al., 2011; Allen et al., 2013). Studies have found that 26.8% of individuals with suicidal ideation will attempt suicide, and more than two-thirds of these individuals will attempt suicide within the first year of suicidal ideation (Ten Have et al., 2013). Therefore, early detection of suicidal ideation is essential to reduce the incidence of suicidal behaviors. Gynecological cancer is one of the major problems hazardous to women’s health. Ovarian cancer is currently one of the three most common gynecological cancers in China, and its mortality rate ranks first among all gynecological cancer (Wang et al., 2021; Zheng et al., 2022). Due to the nonspecific symptoms and the lack of effective screening approaches (Nakao et al., 2020), about 80% of patients have advanced disease when they were diagnosed, with a 5-year survival rates of 20–41% (Torre et al., 2018). Treatment of ovarian cancer often requires complex surgery excision and chemotherapy, each of which would increases morbidity risk (Weaver et al., 2018). After treatment, patients with ovarian cancer confront with a high likelihood of disease recurrence (Coleman et al., 2019). In addition to common cancer-related symptoms, ovarian cancer patients face a number of specific adverse outcomes. These include sexual dysfunction (Rizzuto et al., 2021), fertility loss (Carter et al., 2013), menopausal syndrome (Gernier et al., 2021), body image disorders (Brederecke et al., 2021), etc. The high malignancy and adverse outcomes of ovarian cancer may place patients at high suicide risk (Chen et al., 2022). A study based on the SEER database reported a suicide rate of 13.92 per 100,000 person-years between 1973 and 2017, with a relative risk of 1.9 for women with ovarian cancer compared with the general population (Chen et al., 2022). Extensive literatures have confirmed that the suicide risk of ovarian cancer is highest in patients with gynecological malignancies (Mahdi et al., 2011; Tang et al., 2016; Violette et al., 2019; Chen et al., 2022). A sample based on the Chinese population demonstrated that ovarian cancer patients had suicidal ideation as high as 30.16% (Tang et al., 2016) and remained at the highest risk of suicide over time (Violette et al., 2019; Chen et al., 2022). Therefore, the suicide of ovarian cancer patients deserves attention.

Symptomatic distress refers to the physical and mental suffering or pain of the patients due to the symptoms of the disease itself and/or due to symptoms caused by the treatment (Kuo and Ma, 2002). At present, the main treatment for ovarian cancer patients is surgery, a combination of platinum and paclitaxel-based chemotherapy, radiotherapy, biological therapy and other treatment methods (Penson et al., 2020; González-Martín et al., 2022). During treatment, patients may experience a lot of symptoms, the most common of which are physical symptoms (such as fatigue, pain and hair loss, etc.) (Wan et al., 2015) and characteristic symptoms (such as hot flashes, night sweats and sexual dysfunction) (Wan et al., 2015). These symptoms are often related and interact with each other, and can even aggravate some symptoms, causing great physical and psychological distress to patients (Beck et al., 2005). A growing body of evidence indicates that persistent symptom distress significantly augments the risk of suicidal ideation in cancer patients (Choi et al., 2014; Recklitis et al., 2014; Xu et al., 2020). A survey of 544 hospitalized cancer patients in China found a significant correlation between symptom burden (insomnia and/or loss of appetite) and suicidal thoughts (Xu et al., 2020). The results of a Korean study based on gastric cancer survivors identified physical symptoms such as diarrhea, and hair loss as important risk factors of suicidal ideation (Choi et al., 2014). The effect of symptom distress on suicidal ideation in patients with ovarian cancer has received relatively little attention, nevertheless. In view of the heavy burden of symptoms among ovarian cancer patients and the significant positive correlation between symptom distress and suicidal ideation found in empirical studies (Xu et al., 2020), we hypothesized that symptom distress in ovarian cancer patients could positively predict suicidal ideation in this study.

Depression is very common in ovarian cancer patients. The incidence of depression among patients with ovarian cancer in mainland China was 47.0% (Liu et al., 2017). There is a strong relationship between depression and symptom distress. A study based on a Chinese ovarian cancer population revealed that more severe of symptom distress predicted higher levels of depressive symptoms (Wu et al., 2022). Similarly, a sample from Korea revealed that ovarian cancer patients experienced more depressive symptoms if they were troubled with more physical symptoms (Hwang et al., 2016). Therefore, symptom distress may positively influence depression levels in ovarian cancer patients. In addition, severe depression leads to a constant feeling of sadness and low interest, which will reduce the willingness to live and contribute to suicidal ideation (Sauer et al., 2022). Many studies have confirmed that depression is a key risk factor for suicidal ideation in cancer patients, and the more severe the depressive symptoms, the more likely an individual is to commit suicide (Tang et al., 2016; Kolva et al., 2020; Sauer et al., 2022). This is also true in patients with ovarian cancer (Tang et al., 2016). According to Developmental Psychopathology Model of Suicidal Ideation, the process of suicidal ideation begins with distal risk factors (such as illness and other adverse life events) and gradually develops with proximal risk factors (such as depression) and finally with suicidal ideation (Seguin et al., 2014). This model especially emphasizes that depression is the proximal factor with the strongest association with suicidal ideation and is directly related to suicidal ideation. Other risk factors often contribute to suicidal ideation through the mediating role of depression (Lewis et al., 2021). Therefore, in this study, we hypothesized that depression mediates the relationship between symptom distress and suicidal ideation in ovarian cancer patients.

Not all ovarian cancer patients who are troubled by symptoms, and not all ovarian cancer patients who experience depression will develop suicidal ideation. This suggests that there may be factors that reduce suicidal ideation even in adversity. One of these factors may be the suicide resilience. The American Psychological Association defines resilience to be a person’s ability to recover from adversity, trauma, tragedy, and even major threats (Association, 2014). It is important to note that resilience is a broad concept, and its protective effect against certain adversities depends largely on the specific type of adversities (Sánchez-Teruel et al., 2021). One may have high resilience to some adverse situations, but low resilience to others. To gain a more accurate understanding of individual defenses against suicide, Osman et al. (2004) proposed the concept of suicide resilience, which is considered as perceptions and beliefs that utilize internal and external resources to modulate suicidal thoughts and feelings. Much research has shown a connection between suicide resilience and suicidal ideation, and suicide resilience apparently has a unique protective effect on the development of suicidal ideation (Rutter et al., 2008; Fang et al., 2015; Dhingra et al., 2016). Moreover, studies discovered that cancer patients with high resilience tended to be less depressed (Ristevska-Dimitrovska et al., 2015; Chou et al., 2022). However, low resilience may result in negative mental health indicators (e.g., depression) (Hu et al., 2022). Ovarian cancer patients with low suicide resilience may have difficulty recovering quickly from the stress of cancer, adjusting successfully, and maintaining good mental health. According to the resilience framework, resilience is considered as a dynamic process to mitigate the impact of risk factors on negative outcomes (Kumpfer, 2002). Suicide resilience is of interest because it may keep individuals at lower risk of suicide over their lifetime by buffering various suicide risk factors (Clement et al., 2020). Based on the above research results and theoretical framework, we hypothesized that suicide resilience may moderate the relationship between depression and suicidal ideation. According to Wen and Ye (2014), when the first or second half of the mediation path is regulated by the moderating variable, the mediating effect can also be regulated, that is, there will be differences in the mediating effect at different levels of the moderating variable. Therefore, we also hypothesized that the mediating effect of depression between symptom distress and suicidal ideation was also moderated by suicide resilience.

**Figure 1:**
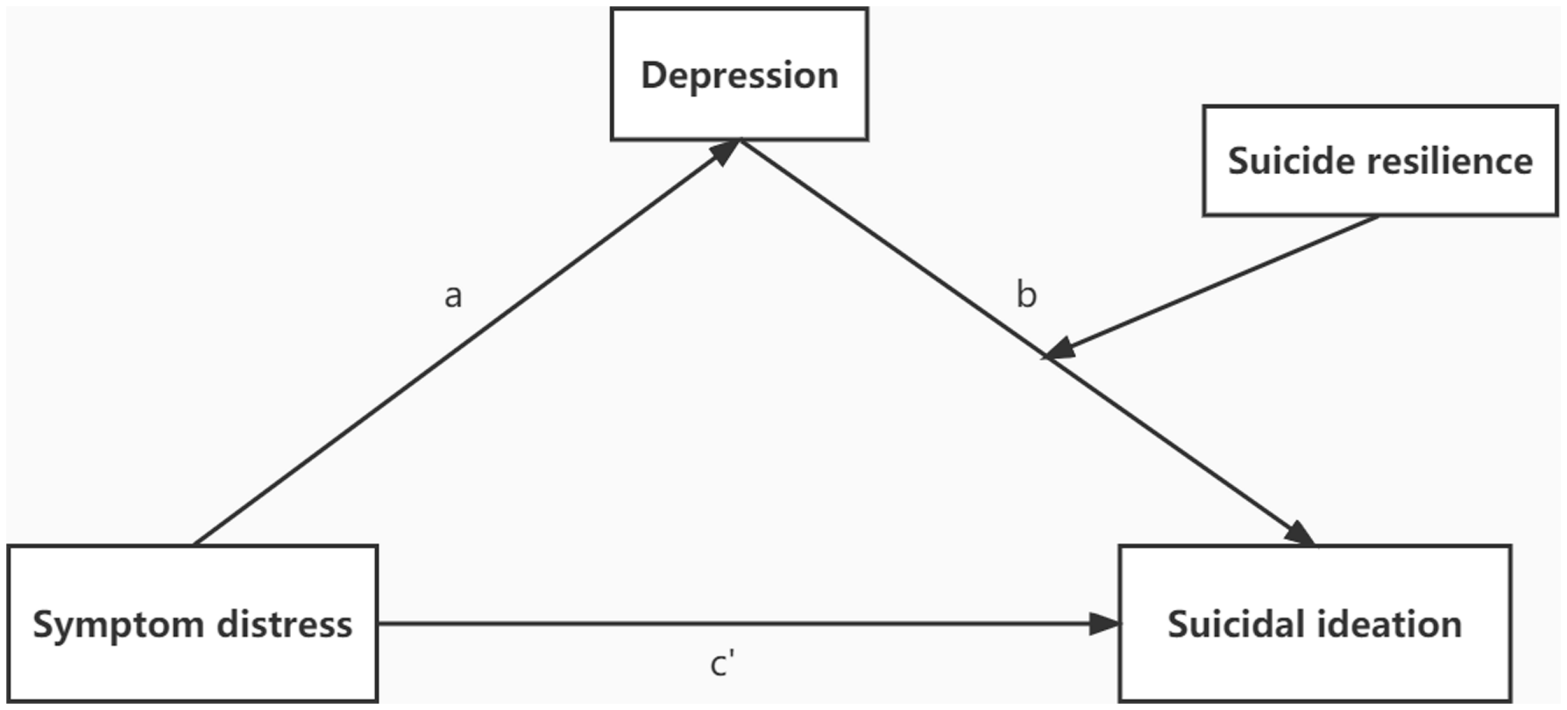
The hypothesized moderated mediation model.

Prior studies have unveiled those individual variables such as symptom distress (Xu et al., 2020), depression (Kolva et al., 2020; Du et al., 2021) and suicide resilience (Dhingra et al., 2016), can strongly predict suicidal ideation in individuals, but no study has confirmed their combined effect on suicidal ideation. Early identification and exploration for the development mechanism of suicidal ideation in ovarian cancer patients have long-term and important significance for the prevention of fatal suicide deaths in the future. It is therefore essential to investigate the influence of factors regulating or intervening in suicidal ideation in ovarian cancer patients. Based on the Developmental Psychopathology Model of Suicidal Ideation and resilience framework, the aim of this research was to explore the relationship between symptom distress and suicidal ideation in ovarian cancer patients, the mediated effect of depression and the moderated effect of suicide resilience. The following hypotheses were proposed (Figure 1): H1: Symptom distress is positively associated with suicidal ideation in patients with ovarian cancer. H2: Depression mediates the association between symptom distress and suicidal ideation. H3: Suicide resilience moderates the relationship between depression and suicidal ideation. And suicide resilience moderates the indirect effect of symptom distress on suicidal ideation through depression. The ultimate purpose is to provide valuable information for prevention strategies of suicide for ovarian cancer patients and prevent loss of life.

## 2. Materials and methods

### 2.1. Design and participants

From March to October 2022, this cross-sectional study was performed in a three Grade.

3A hospital and an oncology specialty hospital in Wuhan, Hubei Province, China. This study has received ethical approval from the Ethics Committee of Huazhong University of Science and Technology (No. S015). Participants were recruited using convenience sampling method. Inclusion criteria: ① patients with histopathological confirmed ovarian cancer who are aware of their disease; ② female and age ≥ 18 years old; ③ patients who experienced chemotherapy; ④ volunteer to participate in and sign written informed consent; ⑤ clear consciousness and can complete the questionnaire with certain comprehension and language skills. Exclusion criteria: ① patients who are critically ill or may have changes of illness at any time; ② there is cognitive dysfunction; ③ a history of mental illness or taking medication for mental illness; ④ unable to communicate or understand the questionnaire items. Power analyses were performed in G * powers with alpha of 0.05, power of 0.95, and moderate effect size of 0.3 to determine adequate sample size (Faul et al., 2009). The required sample size of this study was 191. A total of 241 questionnaires were received, 28 of which were incomplete, and the final sample was 213 after the elimination of the invalid questionnaires, with an effective response rate of 88.38%.

### 2.2. Procedure

A researcher-administered structured questionnaire was applied to collect data by a well-trained master’s student with experience in psychological and suicide research. Before completing the questionnaires, participants signed an informed consent. They were informed of the purpose and procedures of this study and volunteered to take part in. Moreover, the participants were promised that their personal information would be confidentially and anonymously reported. Clean and relatively undisturbed rooms were chosen as survey sites, avoiding treatment and meal time. It took about 15–20 min for each person to complete all the questionnaires. When the participants were confused about the questions in the questionnaire, the researchers would offer explanations and assistance. During the investigation, any participant expressing suicidal thoughts would be informed to the head nurse for further assessment and support.

### 2.3. Instruments

The General Information Questionnaire was developed on the basis of related literature on suicide in ovarian cancer patients (Tang et al., 2016; Chen et al., 2022) and discussions of the research group. A total of 10 demographic variables were included, which were age, marital status, education level, residence, *per capita* monthly household income, employment status, time of diagnosis, cancer stage, metastasis status and pathological type. The Information of cancer stage, metastasis status and pathological type was obtained from the participants’ physicians, and the other information was reported by each participant.

Suicide Resilience Inventory 25 (SRI-25): Suicide resilience was measured using the SRI-25, which is comprised of three-dimensional subscales (Osman et al., 2004). The emotional stability subscale assesses the individual’s ability to resist taking action when they have suicidal thoughts. The external protection subscale evaluates positive perceptions that people are able to get help from others when they were experiencing suicidal thoughts. And the internal protection subscale assesses one’s life satisfaction and overall positive feelings about themselves. The SRI-25 contains 25 items. Each item is scored from 1(strongly disagree) to 6(strongly agree). The total score ranges from 25 to 150. Higher scores indicate greater defenses against thoughts and behavior about suicide. SRI-25 has shown good psychometric characteristics in the Chinese population (Fang et al., 2015). Cronbach’s *α* of the Chinese version of the SRI-25 for the present sample was 0.841.

The Hospital Anxiety and Depression Scale (HADS): The severity of depression was measured by the HADS (Stern, 2014). The scale is composed of two 7-item subscales, of which the depression subscale is applied here. For each item, the score ranges from 0 to 3 points. The total score for depression subscale ranges from 0 to 21. The total score of 7 is the critical value to determine whether there is depression; Grades 8–10 are mild, 11–14 are moderate, and 15–21 are severe depression. The HADS has good reliability and validity in assessing the severity of depression symptoms in different populations, which is a reliable tool for screening depression in general hospitals (Brennan et al., 2010; Luo et al., 2022). Cronbach’s *α* for this sample was 0.832.

The Symptom Module Specific to Ovarian Cancer (TSM-OC): The severity of symptom distress was measured by the TSM-OC (Wan et al., 2015), which was developed with reference to the first part of M. D Anderson Symptom Inventory-Chinese version (MDASI⁃C) (Wang et al., 2004), a classical scale for symptom management of patients with ovarian cancer. TSM-OC has 9 specific symptom items, which are abdominal distension, hair loss, anxiety, hot flashes, night sweats, weight loss, sexual disorder, irritability and feeling weakened female characteristics. Each item is scored from 0 (not at all) to 10 (the most serious), with a total score ranging from 0 to 90. The higher scores denote the more severely distress an individual was troubled with symptoms. This scale was reported to show well internal consistency and tolerance validity (Wan et al., 2015). The present sample showed a Cronbach’s *α* of 0.849.

The Chinese version of Beck Suicide Ideation Scale (BSI-CV): Suicidal ideation was measured by the BSI-CV, which was translated, back translated and revised by Beijing Huilongguan Hospital, and was proved to have good reliability and validity in Chinese adults (Xian-Yun et al., 2010). The scale contains 19 items, among which the first five items are used as screening items. Only if the answer to item 4 (active suicidal thoughts) or item 5 (passive suicidal thoughts) is “weak” or “moderate to strong” (i.e., not 0), items 6–19 are allowed to proceed; Otherwise, the scaling survey is stopped. Each item is scored 0–2 for a total score of 0 to 38. Higher scores denote stronger suicidal ideation and a higher risk of suicide. If items 6–19 are not investigated, the total score is the sum of the first five items’ scores. The Cronbach’s *α* of this scale in this sample was 0.834.

### 2.4. Data analysis

For data analysis, we used EpiData version 3.1 software for data management and IBM SPSS software version 26.0 (IBM Corporation, Armonk, NY, United States). Appropriate descriptive statistics were applied for describing the subjects’ general characteristics. Chi-square tests were applied for statistical comparison in suicidal ideation among Chinese ovarian cancer patients with different general characteristics. Because of the non-normal distribution of the variables in this study, Spearman correlations were calculated to test the relationship among symptom distress, depression, suicide resilience and suicidal ideation. We used Hayes’ PROCESS macro program (Hayes and Scharkow, 2013) to examine mediate and moderate effects. Model 4 of PROCESS macro was to test the mediating effect of depression on the relationship between symptom distress and suicidal ideation. Then, model 14 was used to examine whether the suicide resilience moderated this mediation process. In this study, we chose bias-corrected 95% confidence intervals (CIs) based on 5,000 bootstrap samples to verify the significance of all effects. If the 95% CIs do not contain zero, the effect is considered significant. After standardizing all variables, the mediating and moderating effects were tested. The moderating effect is explained by simple slope test. General characteristics associated with suicidal ideation were controlled as covariates in all effect analyses. We set the level of statistical significance at 0.05 (two-side).

## 3. Results

### 3.1. Common method deviation test

Since the measurements were filled in by self-report, the Harman single-factor method was utilized to check for common method bias of the four scales. The results reported that the eigenvalue of 15 factors were greater than 1. The variation explained by the first factor was 28.534%, which was much less than the critical value (40%). Therefore, significant common method deviations were not detected in this study.

### 3.2. Characteristics of the participants

Two hundred and thirteen female ovarian cancer patients had an average age of 56.84 ± 9.66 (range: 30–81) years. Sixty-three (29.58%) reported suicidal ideation, which comprised 29.6% of the total number in the study. Most ovarian cancer patients were married (85.9%), unemployed (54.0%), reported primary school or lower (33.8%) and lived in rural (45.5%). *Per capita* monthly household income of most ovarian cancer patients was less than 3,000 RMB (40.8%). A total of 54.50% participants were diagnosed with stage III, 71.8% were pathologically diagnosed as serous, 31.0% were diagnosed within 1–3 years and 75.6% had metastasis present. General characteristics were presented in Table 1.

**Table 1:** General characteristics of ovarian cancer patients.

Characteristic (%)	No suicidal ideation (*n* = 150)	Suicidal ideation (*n* = 63)	*p*-value
Age			0.380
<50	29(19.3%)	13(20.6%)	
50–60	72(48.0%)	24(38.1%)	
>60	49(32.7%)	26(41.3%)	
Residence			0.095
Rural	75(50.0%)	22(34.9%)	
Urban	44(29.3%)	27(42.9%)	
Town	31(20.7%)	14(22.2%)	
Marital status			0.177
Married	132(88.0%)	51(81.0%)	
Widowed/separated/single	18(12.0%)	12(19.0%)	
Education level			**<0.001**
Primary school and below	40(26.7%)	32(50.8%)	
Junior high school	56(37.3%)	14(22.2%)	
Technical secondary school or high school	40(26.7%)	5(7.9%)	
College degree and above	14(9.3%)	12(19.0%)	
Employment status			**0.036**
Working	15(10.0%)	8(12.7%)	
Retired	61(40.7)	14(22.2%)	
Unemployed	74(49.3%)	41(65.1%)	
*Per capita* monthly household income (yuan)			**0.001**
<3,000	49(32.7%)	38(60.3%)	
3,000–5,000	57(38.0%)	16(25.4%)	
>5,000	44(29.3%)	9(14.3%)	
Time since diagnosis			0.102
<6 months	46(30.7%)	17(27.0%)	
6–12 months	32(21.3%)	7(11.1%)	
12–36 months	46(30.7%)	20(31.7%)	
>36 months	26(17.3%)	19(30.2%)	
Cancer stage			**<0.001**
Stage I	7(4.7%)	1(1.6%)	
Stage II	35(23.3%)	3(4.8%)	
Stage III	82(54.7%)	34(54.0%)	
Stage IV	26(17.3%)	25(39.7%)	
Metastasis status			**0.003**
Yes	105(70.0%)	56(88.9%)	
No	45(30.0%)	7(11.1%)	
Pathological type			0.273
Serous	107(71.3%)	46(73.0%)	
Endometrioid	22(14.7%)	4(6.3%)	
Mucinous	11(7.3%)	6(9.5%)	
Clear cell	10(6.7%)	7(11.1%)	

Data in bold are statistically significant.

Chi-square tests determined that there were significant differences in ovarian cancer patients’ suicidal ideation by education level (χ^**2**^ = 21.520, *p* < 0.001), employment status (χ^**2**^ = 6.623, *p* = 0.036), *per capita* monthly household income (χ^**2**^ = 14.398, *p* = 0.001), cancer stage (χ^**2**^ = 18.956, *p* < 0.001) and metastasis status (χ^**2**^ = 8.578, *p* = 0.003) (shown in Table 1).

### 3.3. Correlation analysis

The correlation analysis among the four variables were reported in Table 2. Suicidal ideation was positively associated with symptom distress (*rho* = 0.449, *p* < 0.01) and depression (*rho* = 0.463, *p* < 0.01). Symptom distress was also positively relevant to depression (*rho* = 0.519, *p* < 0.01). Suicide resilience was negatively related to suicidal ideation (*rho* = −0.560, *p* < 0.01), depression (*rho* = −0.374, *p* < 0.01) and symptom distress (*rho* = −0.390, *p* < 0.01). These results indicated that the four variables in this study were suitable for further analysis of the moderated mediation and Hypothesis 1 was verified.

**Table 2:** Correlation analysis among variables in ovarian cancer patients.

Variables	M ± SD	Symptom distress	Depression	Suicide resilience	Suicidal ideation
Symptom distress	17.573 ± 9.350	1			
Depression	8.207 ± 5.582	0.519^**^	1		
Suicide resilience	103.319 ± 14.875	−0.390^**^	−0.374^**^	1	
Suicidal ideation	6.244 ± 8.454	0.449^**^	0.463^**^	−0.560^**^	1

^**^p < 0.01.

### 3.4. Testing for moderated mediation effect

Education level, employment status, *per capita* monthly household income, cancer stage and metastasis status were controlled as covariates in all effect analyses. Firstly, model 4 of PROCESS macro was used to check the mediated effect of depression on the relationship between symptom distress and suicidal ideation. When controlling for all covariates, the results (Tables 3, 4) showed that the indirect mediated effect of symptom distress on suicidal ideation was significant. Symptom distress was correlated with suicidal ideation (*B* = 0.417, *t* = 7.799, *p* < 0.001), and the direct predictive effect of symptom distress on suicidal ideation was still significant after depression was included (*B* = 0.258, *t* = 4.455, *p* < 0.001). Symptom distress was a significant positive predictor of depression (*B* = 0.291, *t* = 8.332, *p* < 0.001), and depression had a significantly positively predicted effect on suicidal ideation (*B* = 0.547, *t* = 5.474, *p* < 0.001). In addition to this, the bootstrap 95% CIs for the direct effect of symptom distress on suicidal ideation and the mediated effect of depression were 0.144–0.372 and 0.084–0.244, respectively, which did not include 0 (Table 4), suggesting that symptom distress could not only directly correlate with suicidal ideation, but also predict suicidal ideation through the indirect effect of depression. The direct effect (0.258) and mediation effect (0.159) were responsible for 61.9 and 38.1% of the total effect (0.417), respectively. So, Hypothesis 2 was verified.

**Table 3:** Mediating effect of depression on the relationship between symptom distress and suicidal ideation.

Outcome variable	Predictor variable	*R* ^2^	*F*	*B*	*t*
Suicidal ideation		0.330	16.876^***^		
	Education level			−0.445	−0.706
	Employment status			−0.780	−0.834
	Monthly family income			−1.201	−1.486
	Cancer stage			2.187	2.637^**^
	Metastasis			−0.154	−0.108
	Symptom distress			0.417	7.799^***^
Depression		0.344	17.977^***^		
	Education level			0.229	0.555
	Employment status			−0.617	−1.009
	Monthly family income			−0.951	−1.802
	Cancer stage			1.096	2.024^*^
	Metastasis			−0.740	−0.793
	Symptom distress			0.291	8.332^***^
Suicidal ideation		0.415	20.780^***^		
	Educational level			−0.570	−0.965
	Employment status			−0.443	−0.504
	Monthly family income			−0.681	−0.893
	Cancer stage			1.588	2.024^*^
	Metastasis			0.251	0.187
	Symptom distress			0.258	4.455^***^
	Depression			0.547	5.474^***^

B, unstandardized coefficients. ^*^p < 0.05; ^**^p < 0.01; ^***^p < 0.001.

**Table 4:** Total effect, direct effect and mediating effect.

Symptom distress → suicidal ideation					
	Effect	*SE*	LL 95%CI	UL 95%CI	Proportion
Total effect	0.417	0.053	0.311	0.522	
Direct effect	0.258	0.058	0.144	0.372	61.9%
Indirect effect	0.159	0.041	0.084	0.244	38.1%

SE, standard error; LL95%CI, lower limit of the 95% confidence interval; UL95%CI, upper limit of the 95% confidence interval.

**Figure 2:**
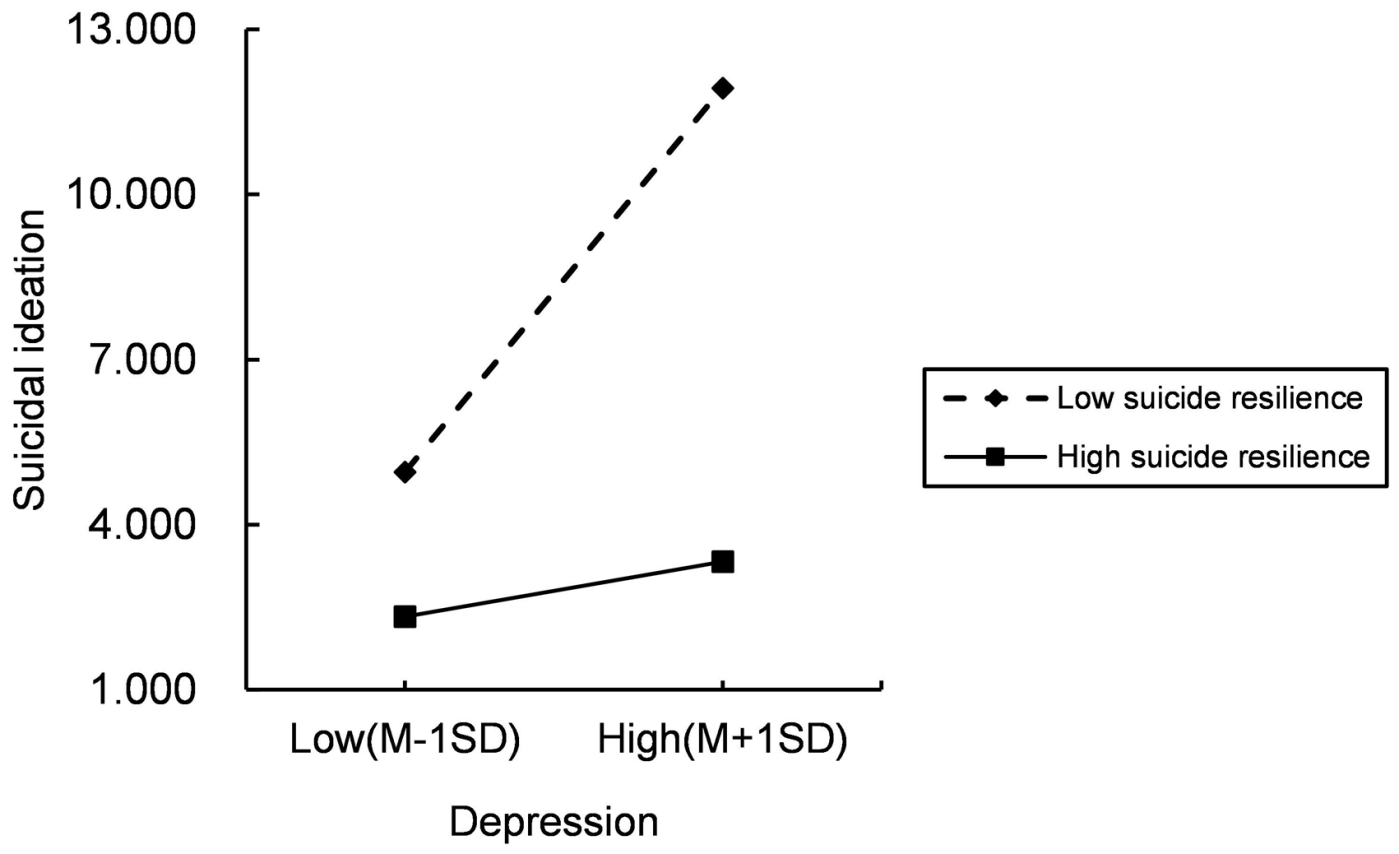
Simple slope analysis.

**Figure 3:**
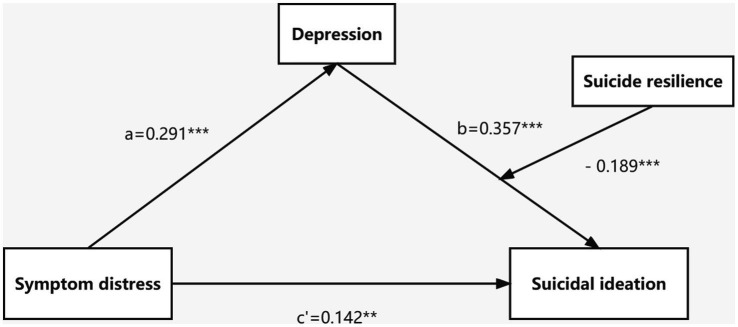
The final moderated mediation model. **p < 0.01; ***p < 0.001.

Secondly, Model14 of PROCESS macro (the latter half of the mediation model path is regulated, which is consistent with the hypothesized model of this study) was applied to check the moderated mediation model under the control of covariates. The results (Table 5) demonstrated that the interaction term (depression × suicide resilience) had a significant moderated effect on suicidal ideation (*B* = −0.180, *t* = −3.360, *p* < 0.001) after adding the suicide resilience into the model, suggesting that suicide resilience could moderate the predictive effect of depression on suicidal ideation. Further simple slope analysis was depicted in Figure 2. Compared to ovarian cancer patients with high suicide resilience, depression had a greater impact on suicidal ideation among ovarian cancer patients with poor suicide resilience, indicating that with the increase of suicide resilience, the predictive effect of depression on suicidal ideation decreased gradually. Moreover, the indirect impact of suicide resilience at different levels (*M*-1 *SD*, *M*, *M* + 1 *SD*) are also shown in Table 6. The indirect effects were significant for ovarian cancer patients with low and average levels of suicide resilience (see Table 6; effect _ow level_ = 0.182, 95%CI: 0.088–0.276; effect_average level_ = 0.104, 95%CI: 0.033–0.175), whereas for ovarian cancer patients with higher suicide resilience, the indirect effect was not significant (see Table 6; effect_high level_ = 0.026, 95%CI: −0.056 – 0.105). That is, with the increase of the level of suicide resilience, symptom distress was less likely to induce suicidal ideation through depression. Thus, Hypothesis 3 was verified. The final model was depicted in Figure 3.

**Table 5:** The moderated mediation model with depression as a mediator and suicide resilience as a moderator.

Outcome variable	Predictor variable	*R* ^2^	*F*	B	*t*
Depression		0.344	17.977^***^		
	Education level			0.229	0.555
	Employment status			−0.617	−1.009
	Monthly family income			−0.951	−1.802
	Cancer stage			1.096	2.024^*^
	Metastasis			−0.740	−0.793
	Symptom distress			0.291	8.332^***^
Suicidal ideation		0.535	25.927^***^		
	Educational level			−0.730	−1.376
	Employment status			−1.213	−1.518
	Monthly family income			−0.819	−1.195
	Cancer stage			1.382	1.932
	Metastasis			−0.619	−0.506
	Symptom distress			0.142	2.607^**^
	Depression			0.357	3.831^***^
	Suicide resilience			−0.189	−5.916^***^
	Depression × Suicide resilience			−0.180	−3.360^***^

^*^p < 0.05; ^**^p < 0.01; ^***^p < 0.001.

**Table 6:** Conditional indirect effects of symptom distress on suicidal ideation at values of resilience.

Suicide resilience	Effect	*SE*	LL 95%CI	UL 95%CI
−14.875	0.182	0.049	0.088	0.276
0.000	0.104	0.036	0.033	0.175
14.875	0.026	0.041	−0.056	0.105

SE, standard error; LL95%CI, lower limit of the 95% confidence interval; UL95%CI, upper limit of the 95% confidence interval.

## 4. Discussion

Approximately 29.58% of ovarian cancer patients reported suicidal ideation in the current sample. This is similar to the incidence of suicidal ideation reported in other ovarian cancer samples (30.16%) (Tang et al., 2016). The study also reported that suicidal ideation in ovarian cancer was highest in the gynecologic cancer group (Tang et al., 2016). Due to the high prevalence and clinical importance of suicidal ideation in ovarian cancer patients, this topic deserves more attention. Although previous studies have discovered that symptom distress is a positive predictor of suicidal ideation, depression as a mediator and suicide resilience as a significant moderator are major strengths of this work. Prior studies (Choi et al., 2014; Xu et al., 2020) have focused only on parts of the moderated mediation model we tested in this work. This study is the first to introduce suicide resilience into the study of the development mechanism of suicidal ideation in ovarian cancer patients and enriches the research on suicide. In this study, we explored the relationship between symptom distress and suicidal ideation, the mediating effect of depression and the moderating effect of suicide resilience in Chinese ovarian cancer patients. The results showed that symptom distress was positively correlated with suicidal ideation in Chinese ovarian cancer patients (Hypothesis 1), and depression played a mediating role in this relationship (Hypothesis 2). In addition, suicide resilience moderated the correlation between depression and suicidal ideation. And it also moderates the indirect effect of symptom distress on suicidal ideation through depression (Hypothesis 3).

### 4.1. Symptom distress and suicidal ideation

According to the results of this research, symptom distress has a strong impact on suicidal ideation in Chinese ovarian cancer patients, which is consistent with previous studies indicating that more severe symptom distress is associated with higher suicide risk (Recklitis et al., 2014; Xu et al., 2020). Ovarian cancer patients experience a range of specific symptoms during treatment, such as abdominal distension, hair loss, hot flashes, night sweats, weight loss, sexual disturbances, irritability, and feelings of diminished femininity (Wan et al., 2015). Due to the diversity and complexity of ovarian cancer symptoms, the extent of symptom distress experienced by patients is often underestimated. And the report of symptoms is not part of routine care, leading to inefficient symptom management and poor quality of life for patients (Sailors et al., 2013). These characteristics and symptoms may cause unique or prolonged suffering for ovarian cancer patients, eventually leading them to think about ending their lives to get rid of their suffering. Our findings suggest that enhanced symptom management will be beneficial in reducing suicidal ideation among ovarian cancer patients.

### 4.2. The mediating effect of depression

Consistent with the hypothesis above, the results confirmed that depression played a mediating role between symptom distress and suicidal ideation in ovarian cancer patients. Thus, depression seems to be one explanatory mechanism that explains why ovarian cancer patients suffering from symptom distress are more likely to develop suicidal ideation. Adverse reactions to chemotherapy could induce emotional instability. Hair loss is a serious symptom in the middle stage of chemotherapy, which has been found to be the most serious and troubling symptom in ovarian cancer (Pozzar et al., 2021). Female patients may feel less feminine or less physically attractive, so they are less likely to engage in social activities, which may reduce their enthusiasm for life to some extent. In addition, a recent study reported that in an ovarian cancer cohort, the highest suicide risk was observed in those who underwent pelvic dissection (Chen et al., 2022). Pelvic dissection is a radical surgical procedure removing organs including the uterus, fallopian tubes and ovaries. The removal of reproductive organs such as ovaries and uteri decrease the female estrogen level, causes vegetal nerve dysfunction, and perimenopausal symptoms such as hot flashes and hyperhidrosis, and may also lead to sexual disorders. Influenced by traditional Chinese concepts, most patients are reluctant to reflect these symptoms truthfully, and medical staff also ignore the assessment and management of the symptoms. In the long run, it will not only affect their marriage and family life, but also make patients appear depressed, anxious and other negative emotions (Stewart et al., 2001). Female patients with ovarian cancer are twice as likely to develop depression symptoms as women without a cancer diagnosis, according to a systematic review and meta-analysis (Watts et al., 2015). Similar to the previous finding (Liu et al., 2017), the proportion of ovarian cancer patients with depressive symptoms was 43.6% in this study. Symptom distress in ovarian cancer patients positively correlated with depression, and current depression was an important factor promoting suicidal ideation. The results of this study supported the developmental psychopathological view of suicidal ideation, which is the result of a chain of multiple factors that develop in a certain order, usually starting with early adversity (distal risk factors), gradually leading to psychological problems (proximal risk factors), and then progressing to suicidal ideation (Seguin et al., 2014). Our results, together with earlier findings, provide evidence of a mediating pathway in which symptom distress increases the risk of depression, which in turn augments the likelihood of suicidal ideation. Therefore, in the whole process of medical treatment, medical staff should pay attention to and early intervene the symptom cluster of ovarian cancer patients to reduce the depression caused by symptoms. It is necessary to strengthen personalized health education, provide comprehensive information support and psychological assistance, and help patients establish a good social support system to prevent the occurrence of suicidal ideation.

### 4.3. The moderating effect of suicide resilience

Consistent with our hypothesis, this study is the first one to show that suicide resilience plays a moderating role between depression and suicidal ideation in ovarian cancer patients. Specifically, for ovarian cancer patients with low levels of suicide resilience, depression may have a significant impact on their suicidal ideation, whereas for patients with higher levels of suicide resilience, depression has no significant effect. Suicide resilience was found to be a protective factor for suicidal ideation in ovarian cancer patients, supporting previous findings (Fang et al., 2015; Dhingra et al., 2016). Furthermore, this study also confirmed that suicide resilience was negatively associated with depression. In terms of tumors, patients with higher resilience are more able to flexibly manage their responses to adversity, successfully adapt to distress, cope with stress, and improve health-related outcomes (Ristevska-Dimitrovska et al., 2015; Chou et al., 2022). Suicide resilience is not a single factor but a series of factors (emotional stability, internal and external protection), which reflects the use of individual resources or abilities to regulate thoughts, feelings or attitudes about suicide (Osman et al., 2004). Resilience Framework (Kumpfer, 2002) and the previous study (Clement et al., 2020) indicated that suicide resilience may reduce individual suicide risk by buffering various risk factors, which remains consistent with the findings of this study. Ovarian cancer patients with high suicide resilience have positive beliefs to regulate their suicidal thoughts or behaviors when facing negative events such as cancer. Internal protection can make oneself satisfied with life and encourage oneself to face setbacks; External protection enables oneself to identify or seek useful external resources when facing difficulties or suicidal thoughts. That is, ovarian cancer patients with higher levels of suicide resilience may have a more positive outlook on life and be able to adapt to the distress of cancer diagnosis, treatment, and symptoms. This resilience may reduce the likelihood that ovarian cancer patients will indulge in depression. Thus, this would trivialize the effect of depression on suicidal ideation. However, ovarian cancer patients with low suicide resilience lack available external resources and lack ability and confidence to solve problems, whereupon, when they are immersed in depression, they cannot cope successfully, ultimately contributing to the development of suicidal ideation. It can be concluded that depression is a risk factor for suicidal ideation in ovarian cancer patients with lower suicide resilience compared with those with higher suicide resilience. In the future research and clinical practice, intervention efforts for individuals experiencing symptom distress or depression could aim to bolster suicide resilience to reduce suicidal ideation in ovarian cancer patients. For example, dialectical behavior therapy (DBT) could be performed, which aims to strengthen strategies for coping with life adversity so that individuals can optimally manage their suffering, thereby reducing the risk of suicide.

### 4.4. Limitations and future directions

Some limitations need to be improved in future studies. Because of the cross-sectional design of this study, causal inferences between the four variables (symptom distress, depression, suicidal ideation, and suicide resilience) cannot be determined. In the future, longitudinal studies with more time points can be designed. Secondly, because all data are based on self-reports from ovarian cancer patients and their recalls of past events vary in accuracy, there may exist a risk of recall bias (Schmier and Halpern, 2004). Ecological momentary assessment (EMA), a method designed to measure and evaluate the process of target behavior, thinking, perception and so on in the real environment, may overcome these defects (Sedano-Capdevila et al., 2021). In the future EMA methodology could improve our understanding and the quality of the data Collected. Thirdly, in this study, we excluded patients with current or lifetime psychiatric diagnoses, which deserved attention and discussion in the field of suicide research. Finally, the sample was recruited from a three Grade 3A hospital and an oncology specialty hospital in Wuhan, Hubei Province, China. The sample sources are limited. Whether and to what extent the results of this study can represent all Chinese ovarian cancer patients remains to be determined, and the findings need to be replicated in other samples.

### 4.5. Implications

The current study has the following implications for suicide prevention and intervention. First of all, symptom distress has a great impact on suicidal ideation in Chinese ovarian cancer patients, which suggests that strengthening symptom management will be beneficial to reduce suicidal ideation. Secondly, depression seems to be a mechanism connecting symptom distress to suicidal ideation in ovarian cancer patients, and alleviating depression may help reduce the effect of symptom distress on suicidal ideation. For example, counseling for ovarian cancer patients to increase their confidence in the face of cancer, helping them back to community life, and providing psychosocial and emotional support may distract them from indulging in negative emotions, thereby reducing their risk of suicide. Finally, the results discovered that suicide resilience was a protective factor buffering the effect of depression on suicidal ideation. This indicates that we should carry out intervention research on suicide resilience in clinical work in the future to improve the ability of individuals to cope with mental health problems and life adversity, so as to avoid the occurrence of suicidal behavior.

## 5. Conclusion

This study contributes to relevant research by highlighting the role of depression and suicide resilience in the relation between symptom distress and suicidal ideation in Chinese ovarian cancer patients. The results suggest that depression is the underlying mechanism linking symptom distress to suicidal ideation, and suicide resilience may be a protective factor buffering this risk process. In suicide prevention and intervention for ovarian cancer patients, these findings may suggest that clinicians should pay attention to the interaction of risk and protective factors.

## Data availability statement

The raw data supporting the conclusions of this article will be made available by the authors, without undue reservation.

## Ethics statement

The studies involving human participants were reviewed and approved by Medical Ethics Committee, Tongji Medical College, Huazhong University of Science and Technology. The patients/participants provided their written informed consent to participate in this study.

## Author contributions

DH: study conception and design. JC: data collection. JX, FC, and KZ: data management and input. JC and YZ: analysis and interpretation of results. JC: draft manuscript preparation. All authors reviewed the results and approved the final version of the manuscript.

## Funding

The study was funded by the National Key Research and Development Plan of China (Grant No. 2020YFC2006000) and National Natural Science Foundation of China (No. 71673100).

## Conflict of interest

The authors declare that the research was conducted in the absence of any commercial or financial relationships that could be construed as a potential conflict of interest.

## Publisher’s note

All claims expressed in this article are solely those of the authors and do not necessarily represent those of their affiliated organizations, or those of the publisher, the editors and the reviewers. Any product that may be evaluated in this article, or claim that may be made by its manufacturer, is not guaranteed or endorsed by the publisher.
